# High-mobility group box-1 protein, lipopolysaccharide-binding protein, interleukin-6 and C-reactive protein in children with community acquired infections and bacteraemia: a prospective study

**DOI:** 10.1186/1471-2334-10-28

**Published:** 2010-02-16

**Authors:** Jana Pavare, Ilze Grope, Imants Kalnins, Dace Gardovska

**Affiliations:** 1 Riga Stradins University, Chair of Pediatrics, Riga, Latvia; 2 Riga Stradins University, Chair of Physics, Riga, Latvia

## Abstract

**Introduction:**

Even though sepsis is one of the common causes of children morbidity and mortality, specific inflammatory markers for identifying sepsis are less studied in children. The main aim of this study was to compare the levels of high-mobility group box-1 protein (HMGB1), Lipopolysaccharide-binding protein (LBP), Interleukin-6 (IL-6) and C-reactive protein (CRP) between infected children without systemic inflammatory response syndrome (SIRS) and children with severe and less severe sepsis. The second aim was to examine HMGB1, LBP, IL6 and CRP as markers for of bacteraemia.

**Methods:**

Totally, 140 children with suspected or proven infections admitted to the Children's Clinical University Hospital of Latvia during 2008 and 2009 were included. Clinical and demographical information as well as infection focus were assessed in all patients. HMGB1, LBP, IL-6 and CRP blood samples were determined. Children with suspected or diagnosed infections were categorized into three groups of severity of infection: (i) infected without SIRS (n = 36), (ii) sepsis (n = 91) and, (iii) severe sepsis (n = 13). They were furthermore classified according bacteraemia into (i) bacteremia (n = 30) and (ii) no bacteraemia (n = 74).

**Results:**

There was no statistically significant difference in HMGB1 levels between children with different levels of sepsis or with and without bacteraemia. The levels of LBP, IL-6 and CRP were statistically significantly higher among patients with sepsis compared to those infected but without SIRS (*p *< 0.001). Furthermore, LBP, IL-6 and CRP were significantly higher in children with severe sepsis compared to those ones with less severe sepsis (*p *< 0.001). Median values of LBP, IL6 and CRP were significantly higher in children with bacteraemia compared to those without bacteraemia. The area under the receiver operating curve (ROC) for detecting bacteraemia was 0.87 for both IL6 and CRP and 0.82 for LBP, respectively.

**Conclusion:**

Elevated levels of LBP, IL-6 and CRP were associated with a more severe level of infection in children. Whereas LBP, IL-6 and CRP seem to be good markers to detect patients with bacteraemia, HMGB1 seem to be of minor importance. LBP, IL-6 and CRP levels may serve as good biomarkers for identifying children with severe sepsis and bacteraemia and, thus, may be routinely used in clinical practice.

## Background

Sepsis in children population is one of the common causes of morbidity and mortality in the word, with > 42 000 cases of severe sepsis in United States annually and millions worldwide [1,2]. Hospital mortality among USA children with severe sepsis was 10.3% [3]. In Latvia between 1995 and 2000, 82 children with sepsis were treated in the only tertiary level hospital in country, with 24.4% of these cases being fatal [4]. Early recognition and prompt initiation of therapy are the most important measures in reducing mortality from sepsis [5-7]. The timely diagnosis of sepsis in children remains difficult due to a variety of reasons: early warning signs and symptoms often are non-specific, the identification process of microorganisms in culture is prolonged.

As a milestone in clinical recognition of sepsis in children became The International Pediatric Sepsis Consensus Conference in 2002 where specific clinical definitions of systemic inflammatory response syndrome (SIRS) and sepsis in children were define [5].

The diagnosis of bacteraemia is still difficult due to prolonged time for microbiological analysis (48-72 hours) and due to the problem that not all bacteria are detectable. However, recent studies have revealed that some inflammatory markers such as the high-mobility group box-1 protein (HMGB1) [8-12], the lipopolysaccharide-binding protein (LBP) [13-16] and the Interleukin-6 (IL-6) [17-20] may be detectable already in the early state of infection and bacteraemia. Whereas the findings in regard HMGB1 were inconsistent in identification of patients with infections and those without [9-12], IL-6 has been suggested to be a suitable early inflammatory marker, with levels correlating well with the severity and prognosis of sepsis [19,20]. Increased serum LBP levels have been reported in neonatal early onset sepsis [14], and is reported to be a better marker in critically ill neonates and children than other markers, such as C-reactive protein, procalcitonin and interleukin-6 (IL-6) [15,16].

Comparatively fewer studies on inflammatory markers in infected children as well in children with bacteraemia have been made, lacking in numbers for LBP, HMGB1. Taking into account the new definition of sepsis in children [21] - systemic inflammatory response syndrome (SIRS) with apparent or confirmed infection - there is a clear need for accurate and rapid laboratory indicators for the early diagnosis of bacteraemia and sepsis in children. The main aim of this study was to compare the levels of HMGB1, LBP, IL-6 and CRP between infected children without systemic inflammatory response syndrome (SIRS) and children with sepsis of different severity levels. Furthermore, a secondary aim was to examine the ability of HMGB1, LBP, IL6 and CRP as markers for early detection of bacteraemia.

## Methods

### Material

All patients admitted to the Children's Clinical University Hospital between January 2008 and May 2009 whose parents agreed to participate were included in this study. The hospital is the only tertiary level children hospital of Latvia that serves a population of ~420,000 children. The inclusion criteria for the study were the suspected diagnosis of an infection by the referring physician and age from 1 week to 18 years. Exclusion criteria were antibacterial therapy within the last 48 h, immunodeficiency, chronic liver or kidney illness, vaccination within 5 days before the start of the illness, any chronic illness that alters CRP levels, congenital metabolic defects, chromosomal anomalies, and use of corticosteroids or immunosuppressant medications.

A total of 140 patients fulfilled the entry criteria (infection) and were enrolled. The percentage of infected patients with sepsis based on SIRS criteria was 74% (n = 104) Patients were classified at the time of inclusion into three following groups of severity of infection: (i) infected without SIRS (n = 36), (ii) sepsis (n = 91) and, (iii) severe sepsis (n = 13). They were furthermore classified according to bacteraemia into (i) patients with bacteremia and/or high possibility of bacteraemia (n = 30) and (ii) patients without bacteraemia (n = 74).

### Definition of infection

SIRS criteria were assessed, taking into account the values of vital signs appropriate to the child's age group, including body temperature, heart rate, respiratory rate and leukocyte count [21]. Classification of the status of SIRS was done by two clinicians without knowing the laboratory results. Sepsis was defined as systemic inflammatory response syndrome (SIRS) in the presence of suspected or proven infection. The diagnose of infection was verified thereafter by positive bacterial culture, tissue strain. Furthermore, evidence of infection included positive clinical findings, imaging or laboratory tests (white blood cells in sterile body fluid, pneumonia in radiographic imaging, petechial or purpuric rash) [21]. For sepsis severity definition the International Pediatric Sepsis Consensus Conference classification was used. Sepsis was defined as severe when the patient had one of the following: cardiovascular dysfunction (hypotension <5^th ^percentile for age, or systolic blood pressure <2SD below normal of age despite >40 ml/kg of isotonic intravenous fluid in 1 hour) or need for vasoactive drug to maintained blood pressure or 2 of the following: unexplained metabolic acidosis, base deficit>5.0 mEq/L, increased arterial lactate > 2 times upper limit of normal, oliguria (urine output < 0.5 ml/kg/h), prolonged capillary refill > 5 sec, core to periphere temperature gap >3°C or acute respiratory distress syndrome (ARDS) as defined by the presence of a PaO2/FiO2 ratio < 300 mm Hg, bilateral infiltrates on chest radiograph, and no evidence of left heart failure or sepsis plus 2 or more organ dysfunctions (respiratory, renal, neurological, hematological or hepatic). Septic shock was defined as sepsis and cardiovascular organ dysfunction. Multiple Organ Dysfunction Syndrome was defined as presence of altered organ function such that homeostasis cannot be maintained without medical intervention [21].

Possible bacteraemia was defined based on the consensus definitions for bloodstream infections in children [22]. The definition includes the presence of SIRS and convincing focus of bacterial infection (pulmonary infiltrates, soft tissue infections, pyelonephritis, osteomyelitis) given a negative blood culture.

### Measurements

A venous blood sample was drawn from each patient under local anesthesia induced by an EMLA patch. All analyzes were made immediately, excepted HMGB, where the samples were processed at frozen at -80°C within 30 min of sampling.

Clinical and demographic data of the patients were assessed and biochemical markers of inflammation (HMGB1, LBP, IL-6, CRP) were determined.

### Laboratory assays

HMGB1 levels were measured with a commercially available enzyme-linked immunosorbent assay (HMGB1 ELISA kit; Shino-Test Corporation, Tokyo, Japan). The measuring range was 1 to 80 ng/ml, the coefficient of variation being <10%. Recovery of HMGB1 in this ELISA was 80-120%.

IL6 and LBP were determined with a chemiluminescent immunometric assay Immulite^® ^2000 (Siemens Medical, Germany). The analytical sensitivity for IL6 was 2 pg/ml and 0.2 μg/ml for LBP.

CRP levels were measured by the latex method (COBAS INTEGRA; Roche professional Diagnostics), the lowest assay sensitivity being 0.085 mg/L. CRP levels <20 mg/L were accepted as normal.

All the laboratory analyses were carried out at the Children Clinical University Hospital (Latvia), except for HMGB 1 which was analyzed in the laboratory of Clinical Immunology and Immunogenetic, Riga Stradins University.

### Ethical considerations

The study protocol was approved by the Central Medical Ethics Committee of Latvia. Each child's parents signed a written consent form. All patients had received the standard of care according to hospital guidelines.

### Statistical analyses

The data was analyzed using SPSS version 17.0 for Windows and Epi Info 2000. The results are presented as numbers (n), frequencies (%), means with respective standard deviation (SD) and as medians with their interquartile ranges (IQR). Differences in continuous variables between different groups of infections were performed using the Kruskal - Wallis test and Mann - Whitney tests as the continuous variables did not follow a normal distribution. Correlation analysis was carried out calculating the Spearman rank coefficient and the respective p-value.

To assess the performance of the selected biomarkers with respect to bacteraemia, receiver operating characteristics (ROC) curves, sensitivity and specificity values were calculated. The 95% confidence interval and p value were reported for the area under the curve (AUC) for the optimal cut-off levels. A p-value of less than 0.05 (two-tailed) was considered statistically significant for all tests.

## Results

### Baseline characteristics of the study sample

The baseline characteristics of the study sample are presented in Table 1. The mean age of was 84.6 months for the infected patients without SIRS, 70.4 months for sepsis patients, and 97.5 months for the severe sepsis patients, respectively.

**Table 1:** Baseline characteristics of the study sample according to severity of infection.

	Infected patients without SIRS (n = 36)	Sepsis patients (n = 91)	Severe sepsis patients (n = 13)
Boys (n)	20	49	11
Girls (n)	16	42	2
Age (months)	84.6 ± 77.0^1^	70.4 ± 69.7	97.5 ± 88.2
			
Number of days of symptoms at hospital admission	3.8 ± 2.4	3.3 ± 2.5	2.9 ± 1.8
Number of day of symptoms at study entry	5.5 ± 3.2	4.5 ± 3.0	3.8 ± 2.0
Treatment time in the hospital (days)	6.3 ± 4.2	8.6 ± 5.9	15.4 ± 13.5

^1^mean ± standard deviation.

The number of infections according to age-groups and infection focus can be found in table 2. The most common infections were upper and lower respiratory track infections as well as gastroenteritis. Bacteraemia was confirmed by 2 separate positive blood cultures. Gram-positive bacteria were identified in 5 patients; Gram-negative bacteria in 3 patients, patients with strongly suspected bacteraemia without microbiological confirmation had pneumonia (11 children), skin/soft tissue infections (6 children), pyelonephritis (4 children), osteomyelitis (1 child).

**Table 2:** Age groups and characteristics of infections among children according to severity of sepsis.

	Infected patients without SIRS^1^ (n = 36)	Sepsis patients (n = 91)	Severe sepsis patients (n = 13)
**Age group**			
0 days -- 1 week	0	0	0
1 week to 1 month	1	4	1
1 month to 1 year	7	23	2
2 to 5 years	11	30	4
6 to 12 years	6	15	0
13 to < 18 years	11	19	6
**Infection focus**			
Upper respiratory tract	13	39	-
Lower respiratory tract	8	27	7
Gastroenteritis	11	13	-
Pyelonephritis	-	4	-
Skin/Soft tissue infection	-	4	3
Osteomyelitis	-	2	1
Meningitis	-	-	2
Occult bacteremia	-	2	-
Cistitis	4	-	-

**Total**	**36**	**91**	**13**

^1^systemic inflammatory response syndrome.

### Differences in levels of HMGB1, LBP, IL6 and CRP between groups of different severity of infection

HMGB1 levels in infected patients without SIRS did not statistically significantly differ from those with sepsis or sever sepsis. In addition there was no statistically significant difference observed in HMGB1 levels between children with sepsis and those with severe sepsis (Table 3). LBP, IL-6 and CRP levels were significantly higher among sepsis patients compared with infected children without SIRS (p < 0.001) and were significantly higher in the severe sepsis group compared with the less severe sepsis group (p < 0.001 for all differences).

**Table 3:** Levels of HMGB1, LBP, IL-6 and CRP in infected children without SIRS, with sepsis and with severe sepsis.

Biomarker	Infected patients without SIRS (n = 36)	Sepsis patients (n = 91)	Severe sepsis patients (n = 13)	p-value^a^
HMGB1 (ng/ml)				
Median	2.5	3.0	3.1	NS
IQR	0.3-5.9	1.0-7.3	1.3-14.3	
p-value^b^		NS	NS	
Lipopolysaccharide--binding protein (μg/ml)				
Median	14.7	26.4	79.7	< 0.001
IQR	8.7-26.0	17.5-42.2	57.8-90.6	
p-value		< 0.001	< 0.001	
Interleukin 6 (pg/ml)				
Median	8.9	32.1	290.0	< 0.001
IQR	4.0--18.8	13.8-68.1	67.9-522.6	
p-value^b^		< 0.001	< 0.001	
C-reactive protein (mg/l)				
Median	12.0	63.0	211.8	< 0.001
IQR	3.1-36.6	27.0-114.5	108.0-318.6	
p-value^b^		< 0.001	< 0.001	

Data presented as median and interquartile range (IQR). SIRS, systemic inflammatory response syndrome. HMGB1, high mobility box-1 protein; LBP, lipopolysaccharide-binding proteine; IL-6, interleukine-6; CRP, C-reactive protein. NS, not significant. ^a^Kruskal-Wallis tests. ^b ^Mann Whitney two independent sample tests, compared with the previous group in the table.

### Differences in levels of HMGB1, LBP, IL6 and CRP between children with and without bacteraemia

There was no statistically significant difference in HMGB1 levels between children patients with and without bacteremia (Table 4). However, LBP, IL-6 and CRP levels were statistically significantly higher in bacteremic patients compared to those without bacteraemia (p < 0.001).

**Table 4:** Levels of HMGB1, LBP, IL-6 and CRP in children with and without bacteraemia.

Biomarker	Infection without bacteraemia (n = 74)	Infection with bacteraemia (n = 30)	p-value^1^
HMGB1^2 ^(ng/ml)	3.0 (1 -- 7.4)^6^	3.2 (1.2 -- 10.9)	ns^7^
LBP^3^(μg/ml)	23.7 (16.6 -- 38.5)	63.4 (28.3 -- 86)	< 0.001
IL-6^4 ^(pg/ml)	21.2 (10.5 -- 46.6)	178.1 (62.3 -- 464.8)	< 0.001
CRP^5 ^(mg/l)	54.5 (16.1 -- 91.1)	212.8 (100.7 -- 247.4)	< 0.001

^1^Mann - Whitney test.;^2^high mobility box-1 protein; ^3^lipopolysaccharide-binding proteine; ^4^interleukine-6; ^5^C-reactive protein; ^6^median and interquartile range (IQR). ^7^not significant.

### Correlations between HMGB1, LBP, IL-6 and CRP in children

No correlations were found between HMGB1 and any of the other three biomarkers (Table 5). LBP correlated well with IL-6 (*r *= 0.688, p < 0.001) and CRP (*r *= 0.741, p < 0.001). In addition, a strong correlation was found between IL-6 and CRP (*r *= 0.632, *p *< 0.001).

**Table 5:** Correlations between HMGB1, LBP, IL-6 and CRP in children with infections

HMGB1 versus marker	Spearman's	*P *value	LBP versus marker	Spearman'sr	*P *value	IL6 versus marker	Spearman's r	*P *value
LBP	0.013	0,899^1^	HMGB1	0.013	0,899	HMGB1	0.115	0,243
IL6	0.115	0,243^1^	IL6	0.688	< 0.001	LBP	0.688	< 0.001
CRP	0.045	0,652^1^	CRP	0.741	< 0.001	CRP	0.632	< 0.001

^1^not significant.

### Diagnostic abilities of HMGB1, LBP, IL6 and CRP in detecting children with bacteremia

**Figure 1:**
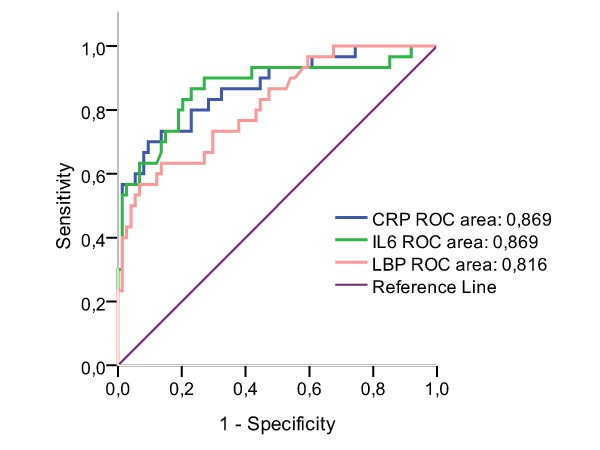
**Receiver operating curves (ROC) of LBP, IL-6 and CRP in detecting bacteraemia in children**. LBP, lipopolysaccharide-binding proteine; IL-6, interleukin-6; CRP, C-reactive protein (p < 0.001).

In receiver operating curve (ROC) analysis for detecting bacteraemic patients, both IL6 and CRP had areas under the curve (AUC) of 0.87 (Figure 1). The 95% confidence interval (CI) was 0.78 - 0.96 for IL6 and 0.79 - 0.95 for CRP, respectively. LBP had an AUC of 0.82 (95% CI 0.73 - 0.91). The sensitivity using a cut-off level of 26.6 μg/ml (LBP), 58.7 pg/ml (IL6) and 97 mg/l (CRP) were 80% in all three markers (Table 6). The corresponding false-positive rates were 55% (LBP), 81% (IL6) and 77% (CRP) for detecting children with bacteraemia.

**Table 6:** Sensitivity and specificity of LBP, IL-6 and CRP according to the optimal cut-off levels in detecting children with bacteraemia.

Marker	Cut-off level	Sensitivity (%)	Specificity (%)
LBP^1^	26.6 μg/ml	80	55
IL-6^2^	58.7 pg/ml	80	81
CRP^3^	97 mg/l	80	77

^1^lipopolysaccharide-binding protein; ^2^interleukin-6; ^3^C-reactive protein

## Discussion

Our study showed that the detectable levels LBP, IL-6 and CRP differ in the earl stage of infection dependent on severity of infection. Furthermore, the detectable levels of these biomarkers differ in children with bacteraemia and those without. In addition, our results showed that LBP, IL-6 and CRP can be used as biomarkers in early detection of bacteraemia.

To date only a few studies are available on the association between SIRS and sepsis in children as the definitions of SIRS and sepsis in children have only recently been adopted.

The inflammatory biomarkers - CRP, IL-6 in children population have been studied comparatively widely, clinical studies of LBP in children are limited and to our knowledge HMGB1 have not been studied in infected children population [14-18]. The previous studies in children population are performed in very restricted patients' populations - mainly neonatal age, which have very age specific physiological conditions with possible influence on results; severe ill patients from intensive care units. Our study, however, included children from all-age groups covering a large spectrum of children with different grades of infections.

The second aim of our study was to facilitate the diagnosis of bacteraemia, as the recognition process of pathogen in the blood is still not perfect. Frequently bacteraemis is strongly suspected without microbiological confirmation and physician institutes the treatment as for bacteraemeic patient. To improve the diagnosis of possible bacteraemia by inflammatory markers, and speed the sequential start of appropriate treatment, we include patients with the high suspicions of bacterial infection (probable bacteraemia) in the group of bacteraemic patients. It is possible that besides gold standard - positive blood culture, the inflammatory biomarkers could speed the diagnosis of bacteraemia. The consequential of it, is the heterogeneity of the bacteraemic patients group, which could be evaluate as the drawback.

HMGB1 has been known from many years as chromosomal protein, but in recent years it has been very intensively investigated as a proinflammatory cytokine [9,23]. In 1999, *Wang et al *[24] found increased levels of HMGB1 in 25 critically ill patients with sepsis, and significantly higher levels in those that succumbed to the disease. Similar findings were reported by Hatada et al. [11] who detected moderately elevated HMGB1 levels in patients with infectious diseases and highest HMGB1 levels in patients who died. Gaini et al. [10] maintained that HMGB1 levels failed to discriminate between internal medicine department patients with infection and those without infection, but HMGB1 levels were significantly higher in patients compared with healthy controls. Similar findings were reported by Sunden-Cullberg et al. [25], who noted high HMGB1 levels in patients with sepsis and septic shock, but found no correlation between HMGB1 concentration and the severity of illness. Angus et al. [26] found that HMGB1 levels did not differ between those with and without sepsis. One possible explanation for these divergent results could be the different laboratory methods used (Western blot and ELISA). Several authors [8,27] have stated that in studies where blotting methods were used, higher levels of HMGB1 in range from 84 ng/ml to over 340 ng/ml were observed [9,25,26]. Using HMGB1 ELISA techniques, markedly lower HMGB1 levels were found. Hatada et al. [11] observed median HMGB1 levels of 4.5 ng/ml] in infected patients. HMGB1 levels of 7.7 ng/ml were measured by Yasuda et al. [28] in infected patients with severe pancreatitis. Gaini et al, [10] in infected patients without sepsis in one of their studies noted median HMGB1 levels of 2.41 ng/ml, and median levels of 3.4 ng/ml in another study [12] which is comparatively close to our HMGB1 result in this patients' group - 2.5 ng/ml. Mean levels of HMGB1 in sepsis patients in our study was 3.0 ng/ml, which are in line with the HMGB1 levels in sepsis patients found in the studies by Gaini et al [10,12]. Furthermore, we did not found a significant difference between HMGB1 levels in infected patients without SIRS and patients with sepsis. These findings are in line with those from Gaini et al [12] as well. In our study using an ELISA technique, median HMGB1 levels were 2.5-3.2 ng/ml which correlate with the lower HMGB1 levels observed in others studies where ELISA technique was used. In contrast to another study [12], we did not found a significant difference between HMBG1 levels in bacteraemic and non bacteraemic patients. This may be due to the fact that in our study not only bacteraemic patients, but also patients with possible bacteraemia were considered as having bacteraemia introducing a misclassification bias. Another possible explanation may be the different age of the children in our study compared to the one conducted by Gaini et al. The HMGB1 is a "late onset" proinflammatory cytokine, but our population was sampled at the early stage of disease. This may be a reason for not finding a significant difference in HMBG1 levels in our study groups.

LBP is a comparatively recently identified protein synthesized mainly in liver. It binds to lipopolysaccharide of Gram negative bacteria, and elevated levels have been seen in infections caused by Gram positive bacteria both in adult [29,30] and children populations [15]. We found a moderate correlation between LBP and CRP, which concurs with the findings of Gaini et al. [12] and partly with their findings in their other publication where a strong correlation was found [31]. We saw a moderate correlation between LBP and IL6, which was also found by Gaini and collegues[31]. In their other study [12], these authors noted a weak correlation between LBP and IL6. In our study LBP levels in sepsis patients (median 26.4 μg/ml) correlates very closely with data from Pavcnik-Arnol [16] where LBP concentrations in SIRS patients with sepsis from population of critically ill neonates were 27.1 μg/ml. Gaini et al. in their studies in sepsis patients accordingly observed LBP levels of 33.5 μg/ml and 63.3 μg/ml [10,12]. In our study patients with severe sepsis median LBP level was 79.7 μg/ml which correlate with Gaini et al. [12] results - LBP levels in severe sepsis patients were 88.7 μg/ml. The levels of LBP were significantly different among infected patients with SIRS and sepsis patients, as well among severe sepsis and sepsis patients which correspondent to the findings from Gaini et al. studies [10,12,31]. In our study LBP levels were significantly different among patients with bacteraemia and without bacteraemia, these results are in line with findings by Gaini et al. [12]. In our study LBP with an AUC of 0.82 performed well on ROC analysis to examine diagnostic abilities in detecting bacteraemia, which correlates with the study by Pavcnik-Arnol et al. [32] where AUC values for LBP in diagnosis of bacterial sepsis were 0.82 in older children, 0.93 in neonates over 48 h and 0.97 in neonates aged under 48 h. LBP in Gaini at al. [12] study in adult patients population with AUC of 0.74 did not perform well in ROC analysis examining its ability to identify bacteraemic patients. In our study using a cut-off level of 26.6 μg/ml, LBP had a sensitivity of 80% and a specificity of 55.4% in diagnosis of bacteraemia which rather correlates with a cut-off level for LBP of 20 μg/ml with 91% sensitivity and 85% specificity by Pavcnik-Arnol [16] in neonates <48 h of age and cutoff 13.3 mg/l for children age.

The levels of the inflammatory cytokine IL6 in our study showed statistically significant differences between infected patients without SIRS and sepsis patients: IL6 was markedly higher in sepsis patients than in patients without SIRS. Overall these results match those of other studies analyzing the levels of IL6 in infected patients without SIRS and sepsis in surgery patients, neonatal age patients, children with acute apendicitis [33-36]. The median levels of IL6 were significantly different among infected patients with SIRS and sepsis patients, and among severe sepsis and sepsis patients, these results correlate Gaini et al. studies [10,12,31]. Median IL6 level for severe sepsis patients in our study was 290 pg/ml, which correlate with the median IL6 levels from Gaini et al. [31], where medium IL6 levels were 199.3 pg/ml for severe sepsis. We found significant difference in IL6 levels among bacteraemic and non bacteraemic patients. IL6 levels 21.2 pg/ml and 178.1 pg/ml accordingly were detected, Gaini et al. [12] observed accordingly 50.3 pg/ml and 178.1 pg/ml in the same patients' groups. We detected a moderate correlation between IL 6 and LBP which is partly consistent with findings from other studies, where in one study a weak correlation was detected and in another a moderate connection was fixed [12,31]. In diagnosing bacteraemia we obtained a cut-off level of 58.7 pg/ml for IL-6 with a sensitivity of 80% and a specificity of 55.4%. Our result is in contrast to results by Pavcnik-Arnol [16] who reported a cut-off of 43.2 pg/ml for bacterial neonatal sepsis diagnosis and Silveira et al. [36] who found an optimal cut-off of 32 pg/ml for neonatal sepsis diagnosis on one side, and a cut-off value of 94.6 pg/ml from Gaini et al. [12] in diagnosing bacteraemia in adult population from another side. In our study on ROC analysis IL-6 performed good, with AUC 0.87, which is quite close to results of Groselj-Grenc [33] - AUC 0.776 for acute appendicitis diagnosis in children, and marginally different from results in Pavcnik-Arnol [16] study - AUC 0.67 for prediction of bacterial sepsis in neonates.

The statistically significant differences in median CRP levels observed between the three groups of children with different severity of infection were in line with the findings of other studies showing higher CRP levels with higher severity of infection in children [31,37].

Naturally, our study had some limitations. The number of patients was rather small due to refusal of study participants (29 patients refused to participate) and the lack of a control group. Furthermore, we included patients with strong suspected (but not confirmed) bacteraemia into the group with bacteraemia which may have caused misclassification bias. However, in clinical work this group would be considered as having bacteraemia and would receive adequate treatment reflecting the real situation in clinical practice.

The issues to be improved are the number of patients included in the study, necessity of a control group consisting of healthy children, and the inclusion of children in the newborn age group. We have recognized the potential problems which we have to meet in future: difficulties to establish the control group - to receive the agreement from parents to take intravenous blood samples from healthy children will be complicated; difficulties to include newborn children in study due to local health-care system (newborn treatment is mainly organized in specialized centers at maternity departments). We have focused on research results that may be useful in future clinical practice for early severe infections diagnosis in children by using effective diagnostic markers.

## Conclusions

The severity of an infection and bacteraemia seem to be associated with increased inflammatory markers levels in children with SIRS. Elevated levels of LBP, IL-6 and CRP were associated with a more severe level of infection in children. Whereas LBP, IL-6 and CRP seem to be good markers to detect patients with bacteraemia, HMGB1 seem to be of minor importance and does not seem to have a diagnostic value in differentiating bacteraemic patients from those without bacteraemia. LBP, IL-6 and CRP levels may serve as good biomarkers for identifying children with severe sepsis and bacteraemia and, thus, may be routinely used in clinical practice.

## Key messages

• The role of HMGB1 as inflammatory cytokine in children has not been thoroughly explored. In our study levels of HMGB1 were not statistically higher in sepsis patients and HMGB1 did not enable to detect bacteraemia in children.

• LBP, IL-6 and CRP levels are associated with the severity of infection in children population.

• IL-6 and CRP are probably the best markers of bacteraemia in children.

## Competing interests

The authors declare that they have no competing interests.

## Authors' contributions

JP planned the study, wrote the protocol, collected and analyzed data, wrote the report. IG was responsible for study planning, collection and analyzing the data, she was involved in practical clinical aspects. IK was responsible for data analysis, he was involved in manuscript revision, DG was involved in study planning, in revising of manuscript. All authors read and approved the manuscript.

## Pre-publication history

The pre-publication history for this paper can be accessed here:

http://www.biomedcentral.com/1471-2334/10/28/prepub
